# A simple method to improve the stability of docetaxel micelles

**DOI:** 10.1038/srep36957

**Published:** 2016-11-11

**Authors:** Lan Zhang, LiWei Tan, LiJuan Chen, XiaoXin Chen, ChaoFeng Long, JinRong Peng, ZhiYong Qian

**Affiliations:** 1 State Key Laboratory of Biotherapy and Cancer Center, West China Hospital, West China Medical School, Sichuan University, Chengdu, 610041, China; 2 Research and Development Department, Guangdong Zhongsheng Pharmacy, Dongguan, 523325, China

## Abstract

Self-assembled polymeric micelles have been widely applied in drug delivery systems. In this study, we found that pH value of micellar system solution was the decisive factor of physical stability. Furthermore, the weak basic solution could maintain the solution clarification for a relative long time. To investigate the stability of polymeric micelles in different pH solutions, the micellar particle size and the docetaxel content remaining in solution were detected at predetermined time points. The crystallographic assay of freeze-drying powder was characterized by an X-ray diffractometer. *In vitro* release results indicated that the PBS had little influence on the sustained-release effect of docetaxel-loaded polymeric micelles (DPM). Besides, the safety of micellar formulation was determined by an MTT assay on HEK293 cells, and the anti-tumor activity was tested on MCF-7 cells. The results demonstrated that DPM adjusted with PBS (DPM (PBS)) was of low toxicity and maintained the effectiveness of docetaxel. *In vivo* antitumor results indicated that DPM (PBS) had better antitumor efficacy than common docetaxel injection (DTX). Thus it was concluded that regulation of micellar solution PH by PBS is a safe and effective method to improve the physical stability of DPM. It might promote the application of micellar formulation in clinical applications.

The chemotherapeutic drugs are restricted by their poor water solubility^1^. Among of them, docetaxel is a such one. Docetaxel is an artificial, semi-synthetic, anticancer drug^2^,^3^, which has shown remarkable effects in treating various solid tumors, such as Kaposi’s sarcoma, metastatic breast cancer, non-small cell lung cancer, stomach cancer, etc^4^,^5^,^6^,^7^. However, because of its poor water solubility, the clinical applications were extremely limited^8^. Micelles were considered to be an advanced drug delivery system for docetaxel^9^,^10^. The polymeric micelles could disperse the hydrophobic drugs in water due to its core-shell structure^11^. Many reports indicated that micelles could passively target agents to tumor tissue via the enhanced permeability and retention (EPR) effect^12^,^13^,^14^,^15^, thus decreasing the toxicity of docetaxel in normal tissue.

In recent years, as a potential drug delivery system for various water-insoluble drugs, micelles have been studied by many researchers^16^,^17^. Lots of micellar formulations of antitumor drugs have also been studied in preclinical and clinical trials. It had been reported that doxorubicin (DOX) containing a biodegradable polymeric micellar system (NK911) was newly developed by using poly(ethylene glycol)-block-poly(aspartic acid) and NK911 was already in Phase I clinical trials^18^,^19^. Also, a paclitaxel-loaded micelle named NK105, which consisted of poly (ethylene glycol)-block-poly (Aspartate) and paclitaxel, was in Phase II clinical trials, which had been prepared by Nippon Kayaku, Japan^20^,^21^,^22^. Then a new doxorubicin formulation (SP1049C) had been investigated to treat advanced adenocarcinoma of the esophagus, which used a combination of two polyethylene oxide polypropylene oxide block copolymers (PEO-PPO), in particular Pluronic L61 and Pluronic F127^23^,^24^,^25^. Genexol-PM (paclitaxel-loaded polymer micelles), studied by Samyang Biopharmaceuticals Corporation and the Korean Breast Cancer Study Group, was currently in Phase II clinical trials for non-small cell lung cancer and recurrent breast cancer in the United States^26^,^27^; PAXCEED, a type of micelle drug for Alzheimer’s, was reached in Phase II clinical trials by Angiotech^28^. Therefore, the polymer micelles are generating much interest in research of delivery systems for hydrophobic drugs.

In spite of many micellar formulations having been studied in clinical trials, none of them have been yet approved by Food and Drug Administration (FDA). This may tell us that the polymer micelles also have lots of defects including stability and insufficient cognition in degradation of materials. However, the factors influencing the micelles’ stability have not been investigated clearly. Since they can be used as a biodegradable carrier in a targeted drug delivery system, finding out what affects the micelles’ stability, and preparing more stable micelles was very fulfilling. Therefore, in this paper, a series of experiments were performed to find the influences of the pH of the environment on the stability of the micelles.

## Results

### Preparation and characterization of docetaxel micelles

**Figure 1:**
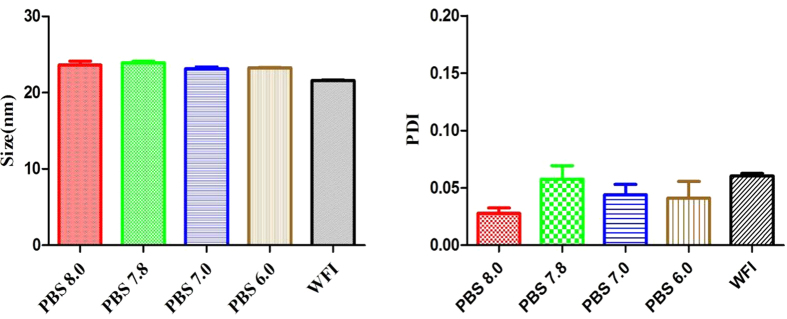
The mean particle size distribution and PDI of DADPM in different pH solution. Error bars represent the standard deviation (n = 3).

In this paper, the docetaxel micelles were prepared by a one-step solid dispersion method. The diblock mPEG-PDLLA, mPEG-PCLA and mPEG-PCL could self-assemble into micelles with docetaxel in water for injection (WFI) at 60 ^o^C. The particle size analysis and the pH of the three kinds of docetaxel copolymer micelles are shown in Table 1. According to a1 to a3, the mean particle size distribution of the docetaxel copolymer micelles was between 10 nm to 30 nm and the PDI was under 0.2. The DADPM was chosen to be characterized in detail. The micelle particle size of DADPM was not affected by the pH of the environment (Fig. 1).

The pH of CDPM, CADPM and DADPM solution was 5.5, 4.3, and 3.7, respectively. Among the three kinds of micelles, the pH of CDPM was the highest and the pH of DADPM was the lowest, All solutions were weakly acid.

**Figure 2:**
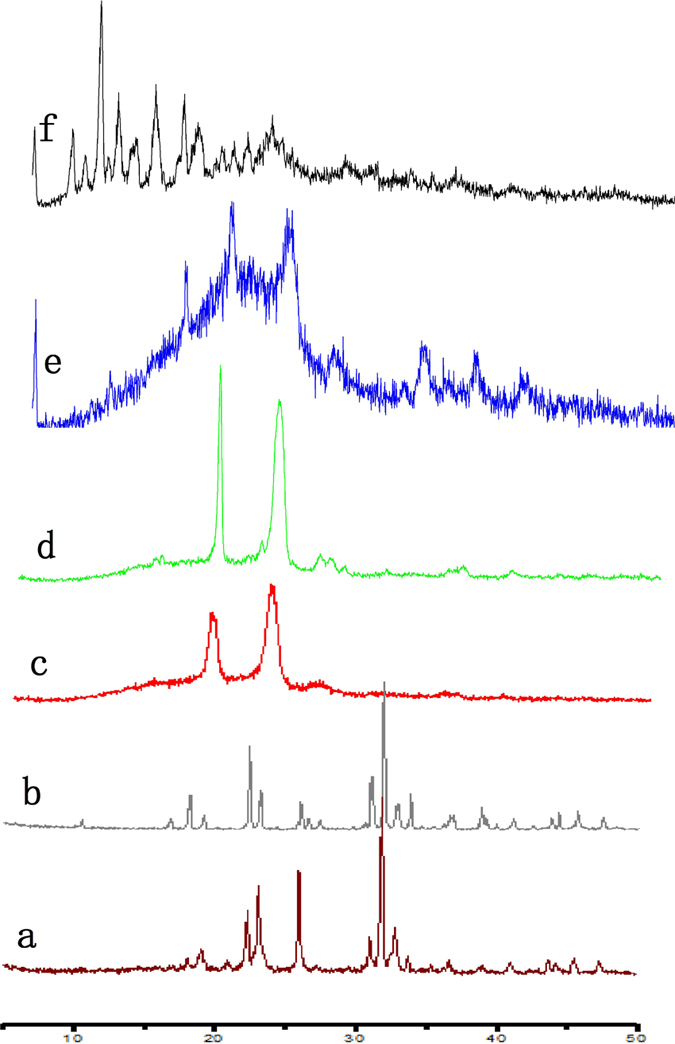
X-ray diffraction spectra of DTX formulations. (**a**) pH8.0 PBS powder; (**b**) DADPM (PBS, PH = 8.0); (**c**) DADMP(WFI); (**d**) Blank mPEG-PDLLA copolymer; (**e**) Physical mixture of docetaxel and mPEG-PDLLA; (**f**) Docetaxel powder.

Crystallographic assay was performed by XRD and the results are shown in Fig. 2. In comparison with the XRD graph of docetaxel powder, DAPM and Doce/DA, the lack of a characteristic diffraction peak in the graph of DADPM indicated that docetaxel was completely wrapped in the core-shell of the micelles. The XRD graph of PH 8.0 PBS powder was the same as the graph of DADPM (PBS, PH = 8.0), which showed that the micelles’ surface was coated with PBS.

### Evaluation of the stability of docetaxel-loaded micelles

**Figure 3:**
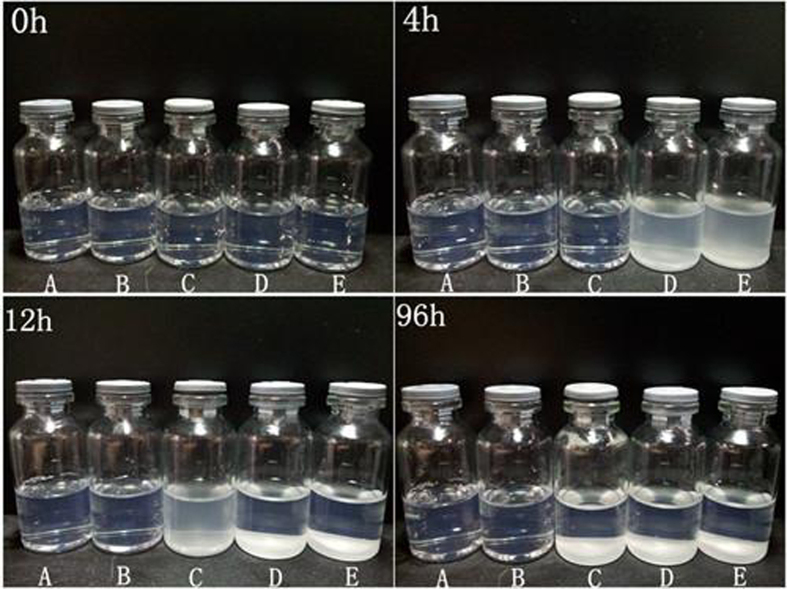
The stability status of different PH of DADPM solution at 0hr, 4 hr, 12 hr and 96 hr after preparation. (**a**) DADPM (PBS, PH = 8.0); (**b**) DADPM (PBS, PH = 7.8); (**c**) DADPM (PBS, PH = 7.0); (**d**) DADPM (PBS, PH = 6.0); (**e**) DADPM (WFI).

With the extension of placement time at room temperature, the different states among them could be observed clearly. According to the macroscopic observation (Fig. 3), the stability of DPM solution with different pH values at 0 hr, 4 hr, 12 hr and 96 hr after preparation is shown. From Fig. 3 we can see that all the micelles’ solutions were clear at the beginning. After 4 hr, in the micellar solution of DADPM (PBS, PH = 6.0) and DADPM (WFI) turbidity appeared, and the others were clear. After 12 hr, the precipitate was formed at the base of the DADPM (PBS, PH = 6.0) and DADPM (WFI), the DADPM (PBS, PH = 7.0) solution had become nepheloid, while the states of DADPM (PBS, PH = 7.8) and DADPM (PBS, PH = 8.0) had no change. After 96 hr, the precipitate had been deposited on the bottom of DADPM (WFI), DADPM (PBS, PH = 6.0) and DADPM (PBS, PH = 7.0) solution while the DADPM (PBS, PH = 7.8), DADPM (PBS, PH = 8.0) solutions were still clear. It could be summarized that the stability of the micellar solution was prolonged with the pH of the solution increasing from 6.0 to 8.0.

**Figure 4:**
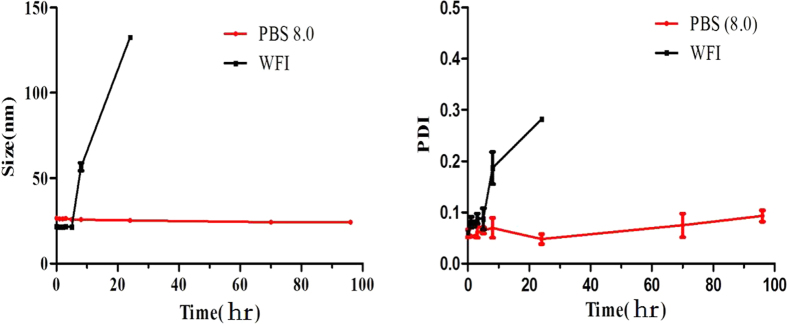
The mean particle size distribution and PDI of DADPM in PBS or WFI at setting time points. Error bars represent the standard deviation (n = 3).

Figure 4 presents the particle size of the micelles in WFI and PBS (PH = 8.0) at the setting time. The mean particle size of DADPM (WFI) increased sharply after 20 hr. Meanwhile, the particle size of the DADPM (PBS, PH = 8.0) was not visibly increased within 9 days, which demonstrated that the PBS (PH = 8.0) maintained the initial form of the micelles for a long time. This may contribute to the micelle circulating in the blood for a long time.

**Figure 5:**
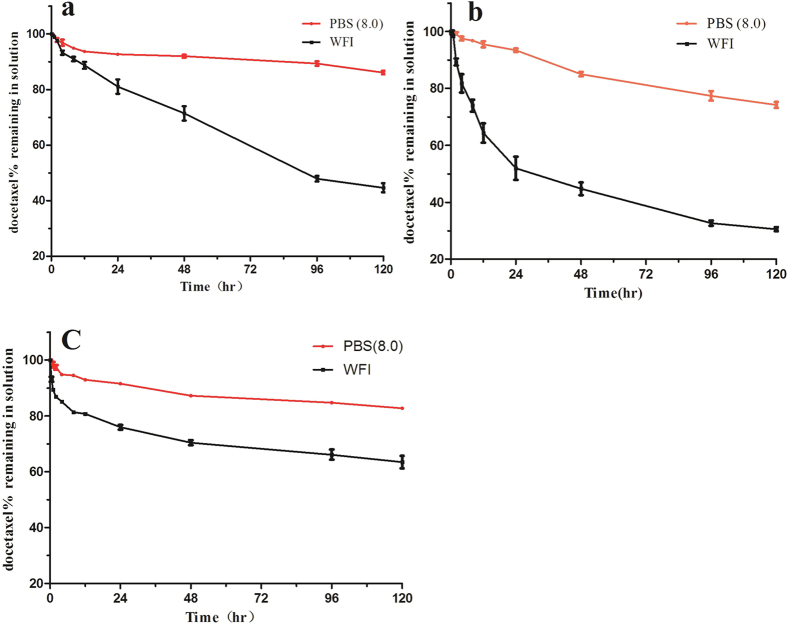
The stability of DPM based on three kinds of polymer in the WFI or PBS (8.0). (**a**) DPM based on mPEG-PCLA; (**b**) DPM based on mPEG-PDLLA; (**c**) DPM based on mPEG-PCL. Initial concentration of docetaxel was 1 mg/ml, placed at room temperature. Error bars represent the standard deviation (n = 3).

The percentage of docetaxel remaining in the sample solutions for CDPM (PBS 8.0), CDPM (WFI), CADPM (PBS, PH = 8.0), CADPM (WFI), DADPM (PBS, PH = 8.0) and DADPM (WFI) placed at 25 ^o^C at 96 hr is shown in Fig. 5. In PBS (8.0), the percentage docetaxel of 3 kinds of micelles remaining in the solution was close to 80%. In WFI, the mean retention of the drug in the solution was 66.1% (CDPM (WFI)), 47.9% (CADPM (WFI)), and 32.7% (DADPM (WFI)) at 96 hr, respectively. It means that the retention of the drug incorporated in the mPEG-PCL was the highest among the formulations and the lowest was mPEG-PDLLA. The drug retention of the three formulations corresponded with the pH tendency at 96 hrs, this phenomenon implied that the stability of the formulation might be dependent on the pH. When we set the pH of solution at 8.0 with PBS, the speed of the micelle reunion slowed down to keep the micelles in the solution instead of as sediment.

**Figure 6:**
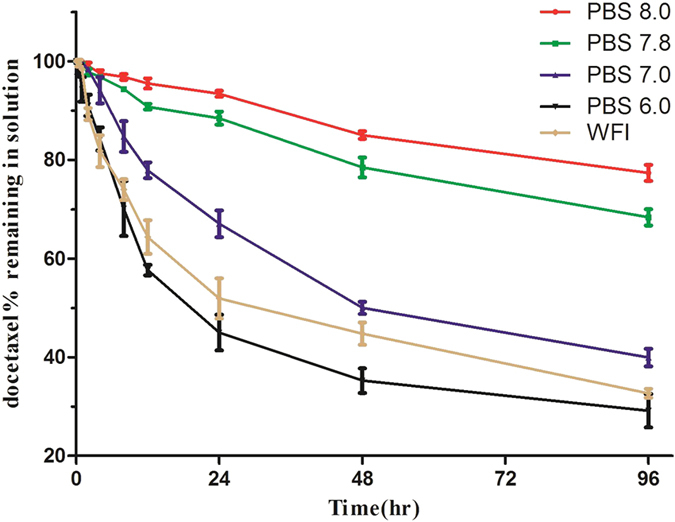
The stability of DADPM in different PH medium. Initial concentration of docetaxel was 1 mg/ml and placed in room temperature within 96 hr. Error bars represent the standard deviation (n = 3).

In addition, the influence of solutions with different pH values on the stability of DADPM was researched. The independent formulation in the PBS solution of pH 8.0, pH 7.8, pH 7.0, pH 6.0 and WFI was sampled and filtered at the setting point in time, and the percentage of drug remaining in the solution was determined through HPLC. The result is shown in Fig. 6, and it demonstrated that as the pH of the solution increased from 6.0 to 8.0, the speed of the micelle reunion became slower. In other words, the stability of the micelles depended on the pH of the solution. Comparing the PBS (6.0) group and WFI group, it indicated that the weak acid solution promoted the release of the drugs from the micelles.

### *In vitro* release study

**Figure 7:**
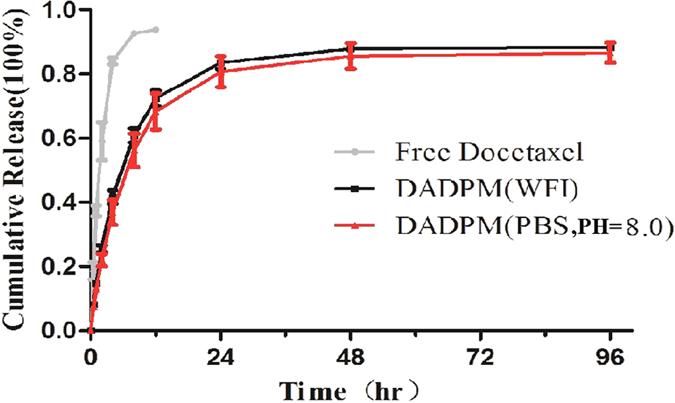
*In vitro* release curve of DADPM(WFI), DADPM (PBS 8.0) and Free Docetaxel solution. Error bars represent the standard deviation (n = 3).

The result of the *in vitro* release profile of free docetaxel, DADPM (PBS, PH = 8.0) and DADPM (WFI) in PBS (PH = 7.4, 37 °C) is illustrated in Fig. 7. In comparison to the micelles, the free docetaxel exhibited a rapid release behavior and more than 90% of the drug was released within 8 hr. It indicated that the PBS buffer made no contribution to the slow-release property of the micelles. This same release process might be ascribed to the fact that the pH of the formulations was adjusted to 7.4 by the *in vitro* release media.

### Cell viability assay

**Figure 8:**
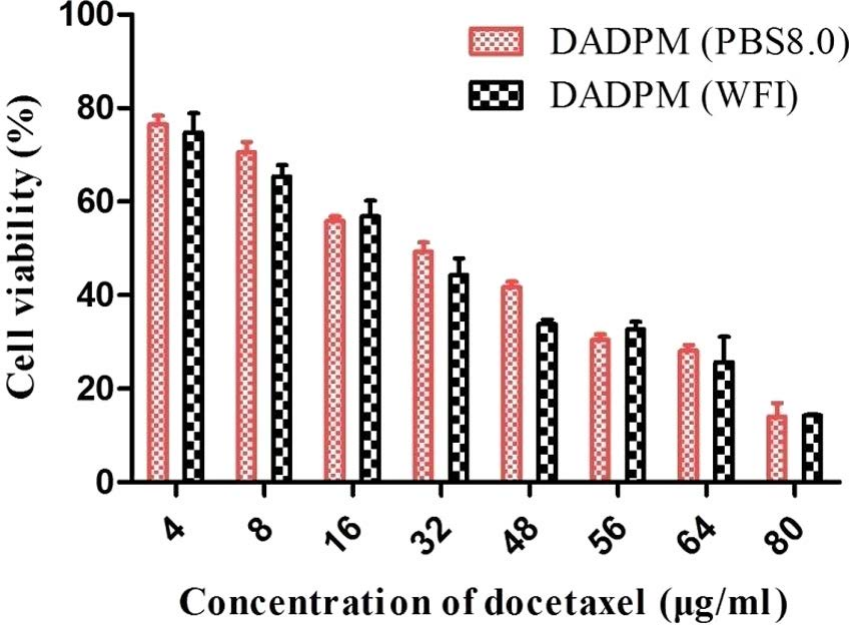
Cytotoxicity *in vitro*. DADPM (8.0) and DADPM(WFI) at different concentrations of docetaxel on MCF-7cells *in vitro*.

The *in vitro* anticancer activity of DADPM (PBS, PH = 8.0) and DADPM (WFI) was compared by the inhibition of MCF-7 cells. In Fig. 8 the 50% inhibiting concentration (IC50) of DADPM (PBS, PH = 8.0) was the same as DADPM (WFI) at about 40 μg/ml. It proved that the DADPM (PBS, PH = 8.0) could fight the cancer cell *in vitro*.

**Figure 9:**
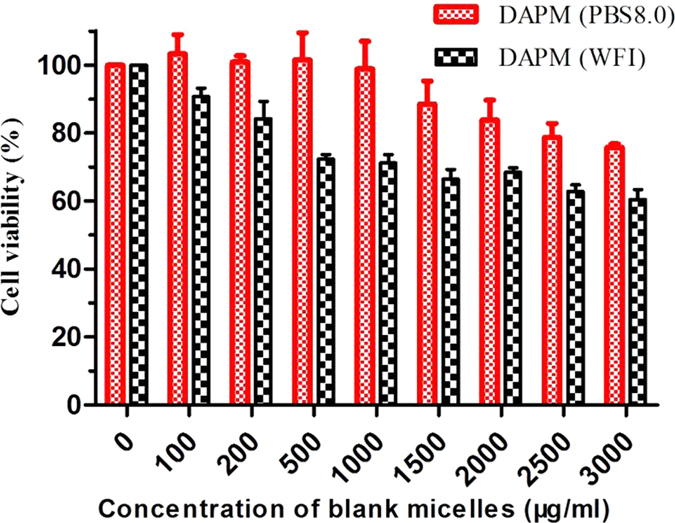
Cytotoxicity *in vitro*. Blank mPEG-PDLLA micelles (PBS 8.0) and blank mPEG-PDLLA micelles(WFI) against HEK293 cells *in vitro*.

The cytotoxicity of DAPM (WFI) and the DAPM (PBS, PH = 8.0) was evaluated using the HEK293 cells. Figure 9 expresses that the IC50 of the two formulations were higher than 3 mg/ml and the cytotoxicity was depended on the concentration of the material. All the results suggest that the DAPM (PBS, PH = 8.0) was low in toxicity and it could be used for intravenous injection.

### *In vivo* antitumor efficacy

To study the antitumor effect of DADPM (PBS, PH = 8.0) and DTX on breast cancer (one of the most common forms of malignancy in women), murine 4T1 breast cancer bearing female BALB/c mice were used as the test animals. After treatment with DADPM (PBS, PH = 8.0) and DTX, the mice’s body weight and tumor volume were evaluated.

**Figure 10:**
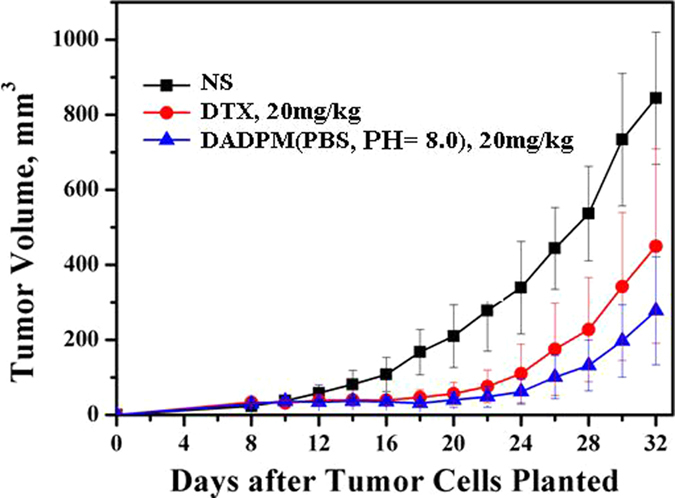
Tumor Volume change of BALB/c mice 4T1 tumor-bearing mice after treatment. Untreated controls received injections of NS (◾), 20 mg/kg of common docetaxel injection (DTX) (⚫) and 20 mg/kg of DADPM (PBS, PH = 8.0) (▴). Each point represents the mean ± S.D. (6 ≤ n ≤ 10).

**Figure 11:**
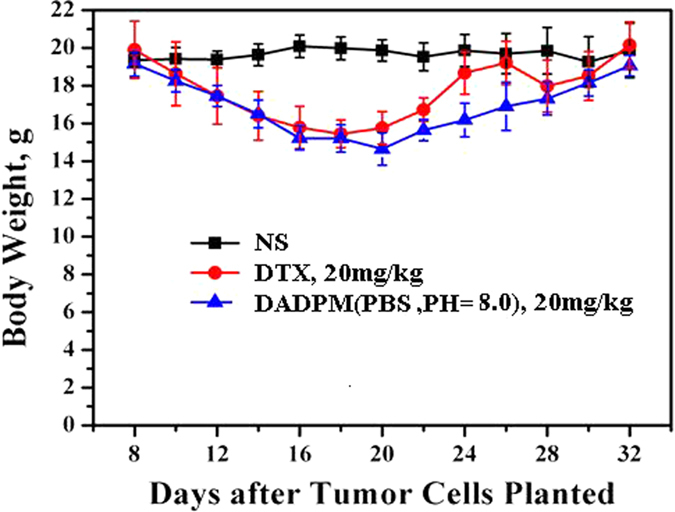
Body weight change of BALB/c mice 4T1 tumor-bearing mice after treatment. Untreated controls received injections of NS (◾), 20 mg/kg of common docetaxel injection (DTX) (⚫) and 20 mg/kg of DADPM (PBS, PH = 8.0) (▴). Each point represents the mean ± S.D. (6 ≤ n ≤ 10).

**Figure 12:**
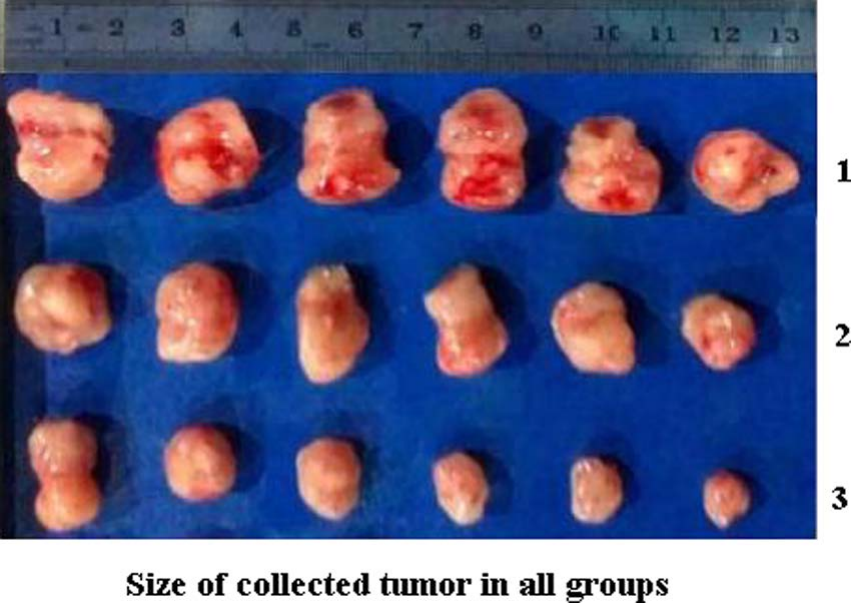
Size of collected tumor in all groups. Untreated controls received injections of NS (1), 20 mg/kg of common docetaxel injection (DTX) (2) and 20 mg/kg of DADPM (PBS, PH = 8.0) (3).

The body weights were monitored every day. After being injected with tumor cells, all the mice had a continuous decrease in body weight. When the treatment began, the tumor volume was calculated, and is shown in Fig. 10. It can be seen that no significant difference emerged after administering the NS intravenously. A similar weight loss occurred with the groups treated with DADPM (PBS, PH = 8.0) and DTX during the whole treatment and recovery period. Figure 11 revealed that the DADPM (PBS, PH = 8.0) was efficient in preventing tumor growth, because the volume of the tumor in the NS group was obviously higher than the other treatment groups. The results of the antitumor experiment show that DADPM (PBS, PH = 8.0) has a better antitumor activity than DTX at the dose of 20 mg/kg. The size of the collected tumor was in accordance with the above results (Fig. 12).

## Discussion

Various chemotherapy drugs occupy a huge area of research because they offer a vast range of possibilities against cancer cells^29^. People have adopted various means to increase solubility, and micelles as one of the effective drug delivery system (DDS) is been investigating by researchers^30^,^31^. Many micellar drugs are being studied for clinical or preclinical use^32^. Among these micellar formulations, Genexol-PM has been used in Korea and the Phase III clinical study has been performed in the United States. However, during the process, the injection must be given within 6 hours, which indicates a limiting stability *in vitro*.

In this paper, we used PBS to adjust the solution to weak alkaline, so that the micellar formulation remained stable for a long time. The results of the determination of the particle size and the drugs remaining in the solution demonstrated this phenomenon, and the cell assay indicated the effectiveness and the low toxicity of the formulation. These characteristics might cause this method to be applied in production and clinical use. From the result of the experiments, the reason that the high pH level could keep the micelle stable might be: polymeric micelles in aqueous solution usually form an aggregate with mPEG as the hydrophilic shell in contact with the surrounding solvent, sequestering the drug in the micelle’s inner core. In micellar solutions, the hydrogen ions, which might intensify attraction and agglomeration of micelles, were often counteracting the negative potential of –OCH_3_ on mPEG, leading to an overall potential drop on the surface of the micelles. The repulsive force among the micelles was also reduced, following an increase in particle size and consequently, the micelles subsided resulting in the instability of the micellar solution. Therefore, the pH value must be adjusted to lower H+ level so as to avoid agglomeration of micelles and eventually improve the stability of preparation *in vitro* and ensure clinical safety. This study has indirectly demonstrated the above standpoint according to the dependency of micelle stability on pH, yet further study will be carried out in the future^33^.

In this study, we found that micelles with PBS (PH = 8.0) have greater advantages over traditional micelles. The settling time of the novel micelle formulation has been significantly longer than the micelle in WFI. Furthermore, its therapeutic efficacy and systemic toxicity was maintained well. In all, changing the pH level of the micelle solution can be an efficient, safe method to improve stability *in vitro*. It’s a marvelous technique for industrial production and clinical application.

According to the results of the *in vivo* antitumor test, we found that DADPM (PBS, PH = 8.0) had a better antitumor activity than DTX at the dose of 20 mg/kg. It might be used in clinical applications. The *in vivo* results indicate that the antitumor efficacy may have positive relation of the stability of micelle solution, we have designed further experiments to study docetaxel micelles’ bioavailability and p-gp activity comparing with common docetaxel injection (taxotere).

Till now, we have prepared a larger sample of docetaxel micelles containing PBS with PH8.0, and the sample reached about 1000 bottle each batch. Meanwhile, through the stability study we found the docetaxel micelles containing PBS with PH8.0 should be kept tight, stored in 2 ~ 8 ^o^C, protected from light. Besides, the antitumor efficacy of the docetaxel micelles containing PBS with PH 8.0 or not is being evaluated in athymic nude mice bearing human lung cancer of H-460, in comparison with Taxotere®.

## Conclusion

In this paper, an excellent method was discovered to improve the stability of the DPM. The data of the experiments show that we have prepared an effective, safe dosage form with palmary physical stability. This novel method provided assistance in the industrial production or the clinical application of the DPM. In short, adjusting the pH of the solution via PBS was a fruitful and feasible method to overcome the instability of the DPM system.

## Materials and Methods

### Materials

The materials used in this article are as follows: Docetaxel was purchased from Xieli Pharmaceutical Co, CHINA. Poly (ethylene glycol)methyl ether (mPEG, Mn = 2000), Stannous octoate ((Sn(Oct)_2_), SIGMA, USA), Acetonitrile for HPLC were purchased from Sigma, USA. Despunlactide (D,L-lactide) was obtained from Daigang Biomateril Co, CHINA. ε-Caprolactone (ε-CL) was purchased from AlfaAesar, USA. anhydrous alcohol (EtOH), sodium phosphate dibasic (NaH_2_PO_4_), Tween80 were purchased from Aladdin, CHINA. Sodium dihydrogen phosphate (NaH_2_PO_4_.2H_2_O) was purchased from KeLong Chemical Co, China. dimethylsulfoxide (DMSO) was purchased from Rgent Chemicals, CHINA. acetic acid was purchased from BODI chemical, CHINA.

### Cell lines and animals

MCF-7 cell lines were incubated in Roswell Park Memorial Institute (RPMI)-1640 Medium (1640) containing 10% bovine serum and 1% antibiotics (penicillin-streptomycin, 100 U/ML) at 37 ^o^C in a humidified atmosphere containing 5% CO_2_. 293 cell lines were incubated in Dulbecoo’s Modified Eagle’s Medium (DMEM) containing 10% fetal bovine serum and 1% antibiotics (penicillin-streptomycin, 100 U/ML) at 37 ^o^C in a humidified atmosphere containing 5% CO_2_. The cell lines in this paper were obtained from the American Type Culture Collection (ATCC). The animals were purchased from the Laboratory Animal Center of Sichuan University (Chengdu, China), which were housed at temperature of 20–22 °C, relative humidity of 50–60% and 12 h light-dark cycles. Free access to food and water was allowed. All animals would be in quarantine for a week before treatment.

All animals care and experimental methods were conducted according to Institutional Animal Care and Use guidelines. All experimental protocols were approved by Medical ethics committee of Sichuang University.

### Synthesis of the diblock copolymers

Poly(ethylene glycol) methyl ether-block-poly(D,L-lactide) (mPEG-PDLLA), Poly(ethylene glycol) methyl ether-block-poly(ε-caprolactone) (mPEG–PCL), and Poly(ethylene glycol) methyl ether-block-poly(ε-caprolactone-co-lactide) (mPEG-PCLA) copolymers were synthesized from different weight ratios of D,L-LA and ε-CL by ring-opening polymerization according to the previous reports^34^,^35^,^36^. The macromolecular weight of the copolymer was characterized by proton nuclear magnetic resonance (^1^H-NMR) spectroscopy and Gel Permeation Chromatography (GPC).

### Preparation of docetaxel micelle

The docetaxel micelles were prepared by a one-step solid dispersion method^37^. Primarily, 5 mg docetaxel and 95 mg copolymer were co-dissolved in 3 ml of absolute ethyl alcohol. Then the solution was evaporated in a rotary evaporator at 60 ^o^C. In this process, the alcohol was evaporated, and homogenous coevaporation was obtained. Subsequently, the mixture was dissolved in the Phosphate Buffer solution (PBS) solution with different PH value. Water was been injected with shaking at 60 ^o^C, and then the micelles would be self-assembled. Then the docetaxel micelle was filtered through 0.22 μm syringe filter (MILLIPORE, BS-GEN-09-01587) to get a clarified solution. Finally the solution was freeze-dried to give a powder of docetaxel polymer micelles.

### Characterization of docetaxel-loaded micelle

#### Determination of particle size of micelles

The particle size distribution of the prepared micelles with different pH values was determined by laser particle size analyzer (Malvern Nano-ZS90). The measurements were carried out after balancing for 2 min at 25 ^o^C, and all the results given were the mean of three test runs.

#### Determination solution PH of co-polymer micelle

The pH of the co-polymer micelle solution was determined by Laboratory pH meter (FE20, METTLER TOLEDO, America) at room temperature, the concentration of copolymer was 19 mg/ml. All the results were the mean of three test runs.

#### X-ray Diffractometer (XRD) analysis

Crystallographic assays were performed on free docetaxel powder, blank mPEG-PDLLA copolymer (DAPM), mPEG-PDLLA-docetaxel-loaded micelles(DADPM), a physical mixture of docetaxel and mPEG-PDLLA (Doce/DA), PH8.0 PBS powder (PBS, PH = 8.0) and docetaxel loaded mPEG-PDLLA micelles with PH8.0 PBS(DAPM (PBS, PH = 8.0)) by X-ray Diffractometer (X’Pert Pro, Philips, Netherlands) using Cu Kαradiation. The data was assembled from step-scan mode from 5 to 50^o^ with a step size of 0.03.

#### Evaluation of the stability of docetaxel-loaded micelle

DAPM in different pH solutions (the pH modifier was PBS) were placed at room temperature. As the precipitate could be seen clearly, the stability of the micelles was evaluated by macroscopic observation.

The particle size of DADPM in two solutions, pH8.0 PBS and the water for injection (WFI), were determined at the different time at 25 ^o^C. The determining method was as same as 2.4.1.

mPEG-PCL-docetaxel micelles (CDPM), mPEG-PCLA-docetaxel micelles (CADPM) and mPEG-PDLLA-docetaxel micelles (DADPM) (docetaxel 1 mg/ml) were left at room temperature after preparation. At appropriate time intervals, 2 ml of the micelle solution was sampled and filtered with a 0.2 μm membrane filter. Then it was diluted with the mixed solution of 49.75% acetonitrile, 49.75% water and 0.05% glacial acetic acid. The concentration of docetaxel remaining in solution was detected by a high performance liquid chromatography (HPLC, Agilent-1260, USA) instrument. The sample concentration was measured by HPLC. The chromatographic conditions were as follows: Chromatographic separation was performed on a reversed phase C_18_ column (Kromasil 100-3.5C18, 150*4.6 mm, E69472). The compositions of the mobile phase were Acetonitrile/ammonium acetate solution (45/55, v/v) at a flow rate of 1 ml/min. Detection was taken on a diode array detector (1260 DAD VL) at the wavelength of 232 nm. Before the measurement, the sample was diluted with acetonitrile.

#### *In vitro* release study

The *in vitro* release behavior of docetaxel from DADPM was developed by a dialysis method as follows: 0.5 ml of DADPM (docetaxel 1 mg/ml) solution was placed in a dialysis bag (molecular mass cut off is 2 KDa), and 0.5 ml of docetaxel dissolved in DMSO (1 mg/ml) was used as a control. The dialysis bag was incubated in 40 ml of phosphate buffer (preheated to 37 ^o^C, pH = 7.4) including Tween80 (0.1%) at 37 ^o^C shaking at about 100 rmp. The media was replaced by preheated fresh media at predetermined time points. The drugs were quantitatively determined by the HPLC method described before. All the results were the mean of three test runs and all data were expressed as the mean ±SD.

#### Cell viability assay

The *in vitro* cytotoxicity of DAPM and DAPM (PBS, PH = 8.0) polymers were evaluated using human embryonic kidney 293 cells (HEK293). Besides, the cytotoxicity of the free drugs and the docetaxel formulation were determined by MTT assays on Michigan Cancer Foundation-7 cells (MCF-7).

In the test, cells were placed in 96-well plates at a density of 3 × 10^3^cells/well in 100 μl of DMEM medium and grown for 24 hr at first. Then the cells were exposed to a series of different concentrations of DAPM, DAPM (PBS, PH = 8.0), free docetaxel, DADPM, and DADPM (PBS, PH = 8.0) for 24 hr. Next, 20 μl of MTT solution (5 mg/ml) was added into each well and incubated for 4 hr. Finally, 160 μl DMSO was added to each well to dissolve the blue crystal that was produced by the viable cell after removing the upper liquid. The optical density (OD) was measured by an ELISA microplate reader (ELX800 Biotek, USA) at 570 nm and the survival of the cell could be calculated.

#### *In vivo* antitumor efficacy

The research protocol was reviewed and approved by the Institutional Animal Care and Treatment Committee of Sichuan University. Female SPF BALB/c mice (8 weeks, 20 ± 2 g) bought by Sichuan University Animal Center (Sichuan, Chengdu, China) were injected subcutaneously in the right abdominal subcutaneous layer with 0.1 ml of cell suspension containing 4T1 breast cancer cells (2 × 10^5^ cells). Treatment was initiated when the tumor reached about 50 mm^3^, which was calculated by the formula *vol* = (*d*^2^ × *a*)/2, where *vol* is volume, *d* is the tumor measurement at the widest point, and *a* is the tumor dimension at the longest point, and this day was designated day 0. On day 0, they were randomly divided into three groups (n = 6 mice/group). A single 0.1 ml dose of NS (as the control group), 20 mg/kg of DADPM (PBS, PH = 8.0) and DTX was respectively administered intravenously on days 0, 1, 2. During this period, the mice were observed continuously for relevant indexes such as weight loss, tumor volume, appetite, diarrhea, cachexia, skin ulceration and toxic deaths. When the treatment began, the tumor volumes and the body weights were calculated. After the last administration, the tumor-bearing nude mice were sacrificed, and tumors were collected and imaged. All the mice were treated humanely throughout the experimental period.

#### Statistical Analysis

All data expressed as mean ± standard deviation were representative of at least three independent experiments.

#### Ethics statement

The methods were carried out in accordance with the approved guidelines.

## Additional Information

**How to cite this article**: Zhang, L. *et al*. A simple method to improve the stability of docetaxel micelles. *Sci. Rep*. **6**, 36957; doi: 10.1038/srep36957 (2016).

**Publisher’s note:** Springer Nature remains neutral with regard to jurisdictional claims in published maps and institutional affiliations.

## Figures and Tables

**Table 1:** The micelles based on three kinds of polymers.

Sample Code	Polymer	Docetaxel: Polymer (w:w)	Size (nm)	PDI	PH
a_1_	MPEG-PCL	5:95	39.3 ± 0.7	0.141	5.5
a_2_	MPEG-PCLA	5:95	25.6 ± 0.4	0.139	4.3
a_3_	MPEG-PDLLA	5:95	21.5 ± 0.2	0.063	3.7
